# Gadolinium labelled nanoliposomes as the platform for MRI theranostics: *in vitro* safety study in liver cells and macrophages

**DOI:** 10.1038/s41598-020-60284-z

**Published:** 2020-03-16

**Authors:** Pavlína Šimečková, František Hubatka, Jan Kotouček, Pavlína Turánek Knötigová, Josef Mašek, Josef Slavík, Ondrej Kováč, Jiří Neča, Pavel Kulich, Dominik Hrebík, Jana Stráská, Kateřina Pěnčíková, Jiřina Procházková, Pavel Diviš, Stuart Macaulay, Robert Mikulík, Milan Raška, Miroslav Machala, Jaroslav Turánek

**Affiliations:** 1 Veterinary Research Institute, Brno, Czech Republic; 2 Central European Institute of Technology CEITEC, Structural Virology, Masaryk University, Brno, Czech Republic; 3 Regional Centre of Advanced Technologies and Materials, Palacký University, Olomouc, Czech Republic; 4 Faculty of Chemistry, Technical University, Brno, Czech Republic; 5 Malvern Instruments, Great Malvern, UK; 6 International Clinical Research Centre, St. Anne’s University Hospital Brno, Brno, Czech Republic; 7 Neurology Department, St. Anne’s University Hospital and Masaryk University, Brno, Czech Republic; 8 Department of Immunology, Faculty of Medicine and Dentistry, Palacký University, Olomouc, Czech Republic

**Keywords:** Reverse transcription polymerase chain reaction, Drug safety, Toxicology, Drug development

## Abstract

Gadolinium (Gd)–based contrast agents are extensively used for magnetic resonance imaging (MRI). Liposomes are potential nanocarrier–based biocompatible platforms for development of new generations of MRI diagnostics. Liposomes with Gd–complexes (Gd–lip) co–encapsulated with thrombolytic agents can serve both for imaging and treatment of various pathological states including stroke. In this study, we evaluated nanosafety of Gd–lip containing PE-DTPA chelating Gd^+3^ prepared by lipid film hydration method. We detected no cytotoxicity of Gd–lip in human liver cells including cancer HepG2, progenitor (non–differentiated) HepaRG, and differentiated HepaRG cells. Furthermore, no potential side effects of Gd–lip were found using a complex system including general biomarkers of toxicity, such as induction of early response genes, oxidative, heat shock and endoplasmic reticulum stress, DNA damage responses, induction of xenobiotic metabolizing enzymes, and changes in sphingolipid metabolism in differentiated HepaRG. Moreover, Gd–lip did not show pro–inflammatory effects, as assessed in an assay based on activation of inflammasome NLRP3 in a model of human macrophages, and release of eicosanoids from HepaRG cells. In conclusion, this i*n vitro* study indicates potential *in vivo* safety of Gd–lip with respect to hepatotoxicity and immunopathology caused by inflammation.

## Introduction

Gadolinium (Gd) –based contrast agents are extensively used for magnetic resonance imaging (MRI). Gd^+3^ forms insoluble phosphate salt in biological fluids and cultivation media. Therefore, Gd^+3^ is used in complexed forms of soluble chelates. These complexes are believed to be stable and non–toxic because of a high stability constant^1^. Recently, accumulation of Gd–based contrast agents has been demonstrated in various organs like kidney, liver and nervous system. It is supposed that toxic effects can be caused by dissociation of Gd ions from chelated complexes^2,3^. Case reports pointed to the induction of nephrotoxicity, hepatotoxicity and neurotoxicity and rare acute adverse reactions to Gd–based contrast agents were also observed in patients^4^.

Liposomes, phospholipid–based vesicles, are widely studied as potential nanocarriers of both MRI contrast agents, including Gd^+3^, and drug molecules, such as thrombolytic agents^1,5^. Therefore, liposomes can serve as diagnostic as well as theranostic agents for imaging and treatment of various pathological states and illnesses such as cancer, ischemic stroke and vasculature of different organs including liver and spleen^6,7^. Thus, liposomes represent a favourable platform for a new generation of targeted diagnostic and theranostic systems^8^.

Liposomes composed of a Gd–chelating lipid, such as 1,2-distearoyl-sn-glycero-3-phosphoethanolamine-N-diethylenetriaminepentaacetic acid (PE-DTPA (Gd)), can be easily modified to capture targeting moieties on their surface using different chemistries including copper–free click–chemistry. Moreover, different surface coating of liposomes including polyethylene glycol, hyaluronic acid and polysaccharides^9–11^ or N-(2-hydroxypropyl) methacrylamide polymers^12^ are suitable to achieve targeting and long–circulation properties of liposomes intended as carriers for contrast agents. By incorporating both PE-DTPA (Gd) and a near infrared dye, liposomal formulations can be used for multimodal imaging^13^. Taken together, liposomes containing Gd–chelating lipids represent a platform for the development of theranostics with a potentially broad range of applications.

The biodistribution of Gd^+3^ chelates formulated into liposomes, or other biocompatible nanocarriers, can significantly differ from low molecular weight Gd^+3^ complexes after their administration in patients. Hepatocytes and immune cells are the main off–target cells when nanoparticle based theranostics are administered to the blood circulation to visualize structures like thrombi or obstruction in blood vessels. Therefore, a more detailed study of potential toxicological effects of lipid–based Gd^+3^ formulations on different organs and cell types including liver cells and immune cells is of importance for a future clinical application in patients.

In this study, nano–safety of MRI imaging liposomal platform containing Gd^+3^ chelator lipid PE-DTPA, 1,2-distearoyl-sn-glycero-3-phosphocholine (DSPC), and cholesterol (5/65/30 molar % of lipid) were tested in *in vitro* cell culture models for potential toxicological effects. For this purpose we used a complex system of general biomarkers of toxicity, including cytotoxicity, changes in sphingolipid metabolism, induction of early response genes^14^ and markers of heat shock response^15^, endoplasmic reticulum (ER) stress^16^, oxidative stress^17^, DNA damage response^18^, inflammation and modulation of xenobiotic metabolizing enzymes.

## Results

### Preparation and characterization of Gd–liposomes

**Figure 1:**
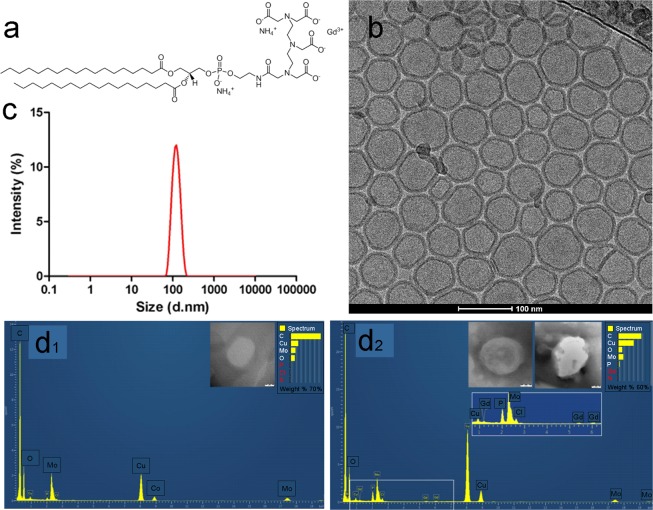
Characterization of Gd–liposomes. Chemical structure of PE-DTPA (Gd) lipid (**a**). Unilamellar structure of monodisperse Gd–lip was confirmed using cryo–TEM (**b**) (*note enhanced contrast of liposomal membrane and resolution of the inner and outer phospholipid layer in liposome membrane owing to Gd atoms presented in phospholipid PE-DTPA (Gd)*. The size distribution of Gd–lip measured by DLS method (**c**). Detection of Gd in liposomes by TEM–EDX: Spectrum of control liposomes (**d**_**1**_) and Gd–liposomes (**d**_**2**_). Detail of Gd peaks is in the insert (**d**_**2**_).

Gadolinium containing liposome (Gd–lip) preparations were represented by unilamellar liposomes with low polydispersity as demonstrated by dynamic light scattering (DLS) and cryo–transmission electron microscopy (TEM) methods. The structure of liposomes and their size distribution are presented in Fig. 1. Incorporation of PE-DTPA (Gd) lipid enables resolution of outer and inner lipid bilayer in liposome phospholipid membrane by cryo–TEM (Fig. 1b). Based on cryo–TEM data, the thickness of liposomal bilayer was 6.68 ± 0.3 nm for liposomes containing PE-DTPA (Gd) lipid (thickness means the distance of Gd atoms on both sides of the bilayers). The bilayer thickness in liposomes without Gd is about 4.6 ± 0.3 nm (distance of phosphorus atoms on both sides of the bilayer). Association of Gd with liposomes was confirmed by TEM equipped with energy dispersive spectroscopy system (EDX) detector (Fig. 1d_2_).

Values of ζ–potential reflected surface modification of liposomes by metallochelating lipids with DTPA head group. Plain liposomes exerted nearly neutral values of ζ–potential (−3 ± 2.2 mV). Liposomes modified by PE-DTPA (without complexed Gd^+3^) are negatively charged (−62.3 ± 1.2 mV), which is reflected by a highly negative value of ζ–potential. Complexing of the Gd^+3^ cations results in a slight increase of ζ–potential (−57.6 ± 1.9 mV) because of binding of carboxyl group ligands to Gd^+3^ and forming a stable complex (Table 1).

**Table 1:** Characterization of Gd–liposomes.

Parameter	Gd–liposomes
Size distribution (zeta–average)	113 +/− 0.5 nm
Polydispersity index (PDI)	0.04 +/− 0.01
Size distribution — Intensity	119 +/− 0.7 nm
Size distribution — Volume	108 +/− 0.2 nm
Size distribution — Number	91 +/− 0.5 nm
Total phospholipid conc. (Stewart assay)	0.4538 mg/ml
Particle density	1.59E+11 particles/ml
Concentration of Gd (inductively coupled plasma optical emission spectrometer)	0.0392 mM
ζ–potential (10 mM Tris, pH 7.4) Gd–liposomes	−57.6 +/− 1.9 mV
ζ–potential (10 mM Tris, pH 7.4) Control Liposomes	−62.3 +/− 1.2 mV

### Cytotoxicity test of Gd–liposomes in liver cells

The cytotoxicity of Gd–lip was tested by the neutral red (NR) uptake assay in human liver cancer HepG2 cells, liver progenitor (non–differentiated) HepaRG cells and HepaRG cells differentiated into two cell populations, hepatocyte–like and biliary epithelial cells, which is a liver model closest to primary hepatocytes and well–established model for drug–induced human hepatotoxicity studies^19^. The concentration of Gd ranged between 1 μM and 100 μM, and no cytotoxicity was observed up to 72 h of exposure (Supplementary Fig. S1). Neither control liposomes (ctrl–lip), having the same lipid composition, nor clinically used MRI contrast agent Gd–DOTA (gadolinium (III) 1,4,7,10-Tetraazacyclododecane-1,4,7,10-tetraacetate), affected the viability of the liver cells in this assay.

### Gd–liposomes are localized to lysosomes in differentiated HepaRG cells

**Figure 2:**
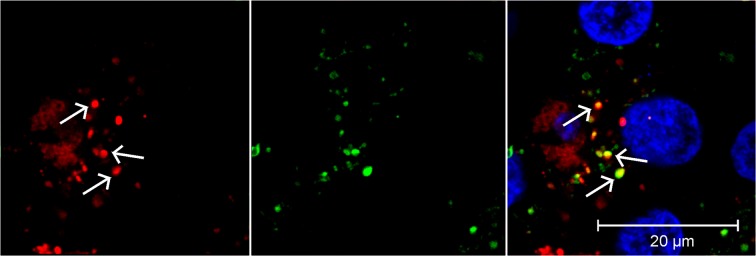
Confocal microscopy of differentiated HepaRG cells. The cells were exposed to Gd–lip labelled with Lyssamine–Rhodamine (red) for 4 h. The lysosomes and nuclei were visualized with Lysotracker Green and Hoechst 33342, respectively. Arrows point to liposomes (red) localized in lysosomes (green) in the merged picture.

In order to track the intracellular fate of Gd–lip by confocal microscopy, red fluorescent lipid, 1,2-dioleoyl-sn-glycero-3-phosphoethanolamine-N-(lissamine rhodamine B sulfonyl) (ammonium salt; 18:1 Liss Rhod PE), was incorporated into the liposomes that were subsequently used for exposure of differentiated HepaRG cells. After 4 h, red liposomes were observed inside biliary cells and co–localized with Lysotracker, indicating lysosomal localization (Fig. 2).

### Gd–liposomes enter into the cells but do not affect sphingolipid metabolism

**Figure 3:**
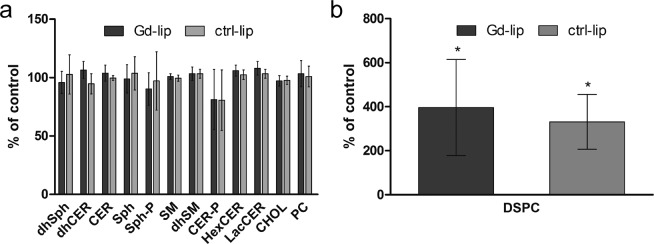
Changes in the amount of sphingolipid species (**a**) and phospholipid DSPC (**b**) as measured by LC/MS–MS. Differentiated HepaRG cells were exposed to ctrl–lip or Gd–lip for 24 h. The results are expressed as mean ± s.d. of three independent experiments. The means are significantly different from negative control (untreated cells) at *p <0.05. Abbreviations: dhSph, dihydrosphingosine; dhCER, dihydroceramides; CER, ceramides; Sph, sphingosines; Sph-P, sphingosine-1-phosphate; SM, sphingomyelins; dhSM, dihydrosphingomyelins; CER-P, ceramide-1-phosphate; HexCER, hexosylceramides; LacCER, lactosylceramides; CHOL, cholesterol; PC, phosphatidylcholine; DSPC, 1,2-distearoyl-sn-glycero-3-phosphocholine.

In order to reveal possible adverse effects of Gd–lip that may precede visible manifestation of cytotoxicity, we exposed differentiated HepaRG cells to Gd–lip or ctrl–lip for 24 h and determined relative changes of sphingolipids, cholesterol, and phosphatidylcholine by liquid chromatography/tandem mass spectrometry (LC/MS–MS) analysis. We found no statistically significant changes in the lipid content (Fig. 3a). The amount of the main liposome–forming lipid DSPC, was increased up to 300% in cells exposed to both Gd–lip and ctrl–lip compared to control cells (Fig. 3b), thus proving that liposomes enter into the cells regardless of the Gd content.

### Gd–liposomes do not change expression of stress–related genes

The toxicity of Gd–lip was further evaluated using a complex system of general biomarkers of toxicity, including the induction of early response genes, heat shock stress, ER stress, oxidative stress, and DNA damage responses and modulation of xenobiotic metabolizing enzymes. Differentiated HepaRG cells were exposed to ctrl–lip or Gd–lip for 24 h and changes in mRNA expression were measured by reverse transcription polymerase chain reaction (RT–PCR).

**Figure 4:**
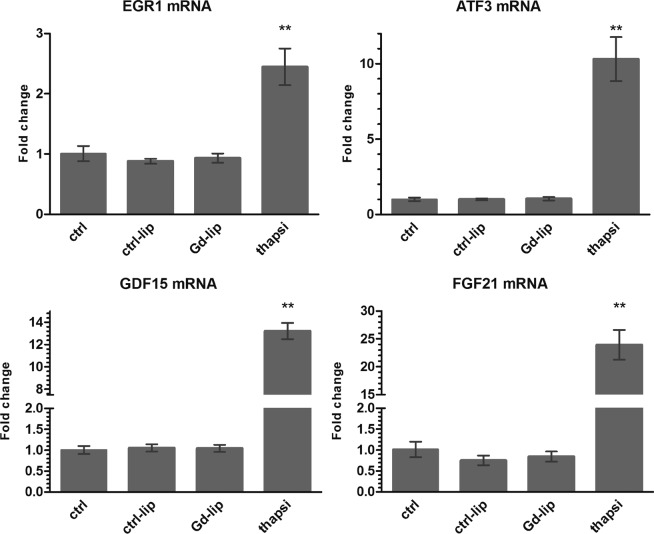
Induction of early response genes: ATF3, EGR1, GDF15, and FGF21. Differentiated HepaRG cells were exposed to ctrl–lip or Gd–lip for 24 h and relative mRNA levels were determined by RT–PCR. 24h exposure to 3 μM thapsigargin was used as a positive control. The results are expressed as means ± s.d. of four independent experiments. The means are significantly different from negative control at **p < 0.01.

At first, we employed a set of 4 genes, early growth response–1 (EGR1), activating transcription factor 3 (ATF3), growth differentiation factor 15 (GDF15) and fibroblast growth factor 21 (FGF21), that have been previously suggested as markers of early toxicity^14^. Changes in their expression were shown to be induced far before the manifestation of toxicity is observable at the cellular level. The genes were identified in primary human and rat hepatocytes and can predict liver and kidney pathology *in vivo*. In our study, neither ctrl–lip nor Gd–lip changed ATF3, EGR1, GDF15 and FGF21 mRNA levels in contrast to thapsigargin, a calcium homeostasis disrupting substance, used as a positive control (Fig. 4).

**Figure 5:**
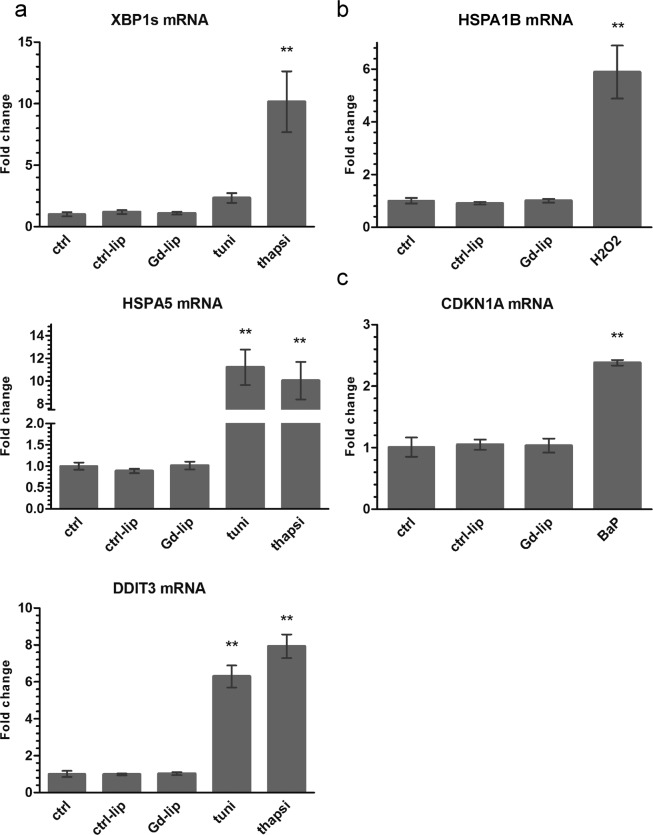
Expression changes of genes involved in unfolded protein response (**a**), heat shock protein response (**b**) and DNA damage response (**c**). XBP1s, HSPA5, DDIT3, HSPA1B and CDKN1A mRNAs were measured by RT–PCR in differentiated HepaRG cells exposed to ctrl–lip or Gd–lip for 24 h. 24h exposure to 3 μM thapsigargin (thapsi), 4 μg/ml tunicamycin (tuni), or 10 μM BaP and 5h exposure to 400 μM H_2_O_2_ were used as positive controls. The results are expressed as mean ± s.d. of four independent experiments. The means are significantly different from negative control at **p < 0.01.

Next, we determined mRNA levels of heat shock response and ER stress markers, the latter identified as induction of unfolded protein response (UPR) genes — spliced X–box binding protein 1 (XBP1s), heat shock protein A (Hsp70) member 5 (HSPA5; also known as BiP) and DNA damage inducible transcript 3 (DDIT3). No expression changes of the UPR–related genes (Fig. 5a), nor stress–inducible heat shock protein gene, HSPA1B (also known as HSP70–2) (Fig. 5b), were detected at the mRNA level in cells exposed to Gd–lip or ctrl–lip. The positive controls were cells exposed to prototypical chemical inducers of cellular stress, thapsigargin, tunicamycin, benzo[a]pyrene (BaP), or H_2_O_2_. Additionally, neither Gd–lip nor ctrl–lip liposomes caused DNA damage, as the relative mRNA level of cyclin–dependent kinase inhibitor 1A (CDKN1A), the p53 target gene and marker of genotoxicity and DNA–damage response activation, remained unchanged (Fig. 5c).

**Figure 6:**
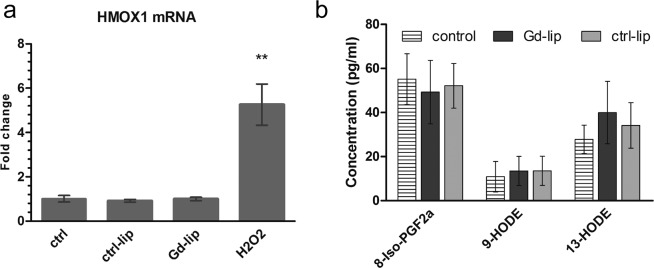
Oxidative stress evaluation. Level of HMOX mRNA was determined by RT–PCR (**a**). Concentration of 8-isoPGF_2α_, 9-HODE and 13-HODE (pg/ml) in culture medium was measured by LC/MS–MS (**b**). Differentiated HepaRG cells were exposed to ctrl–lip or Gd–lip for 24 h. 5h exposure to 400 μM H_2_O_2_ was used as positive control. The results are expressed as mean ± s.d. of four independent experiments. The means are significantly different from negative control at **p <0.01.

In order to reveal potential induction of oxidative stress in cells exposed to Gd–lip, we detected mRNA level of heme oxygenase–1 (HMOX–1), an antioxidative enzyme–encoding gene. Neither Gd–lip nor ctrl–lip changed the HMOX1 mRNA levels (Fig. 6a) in contrast to H_2_O_2_, used as a positive control. Furthermore, liposomes did not change the concentrations of lipid peroxidation products, 8-iso-prostaglandin F_2α_ (8-iso-PGF_2α_) and hydroxyoctadecadienoic acids (HODEs) (Fig. 6b), arisen from oxidation of arachidonic and linoleic acid, respectively. Taken together, it was proven that neither Gd–lip nor ctrl–lip induced oxidative stress and lipid peroxidation in HepaRG cells.

**Figure 7:**
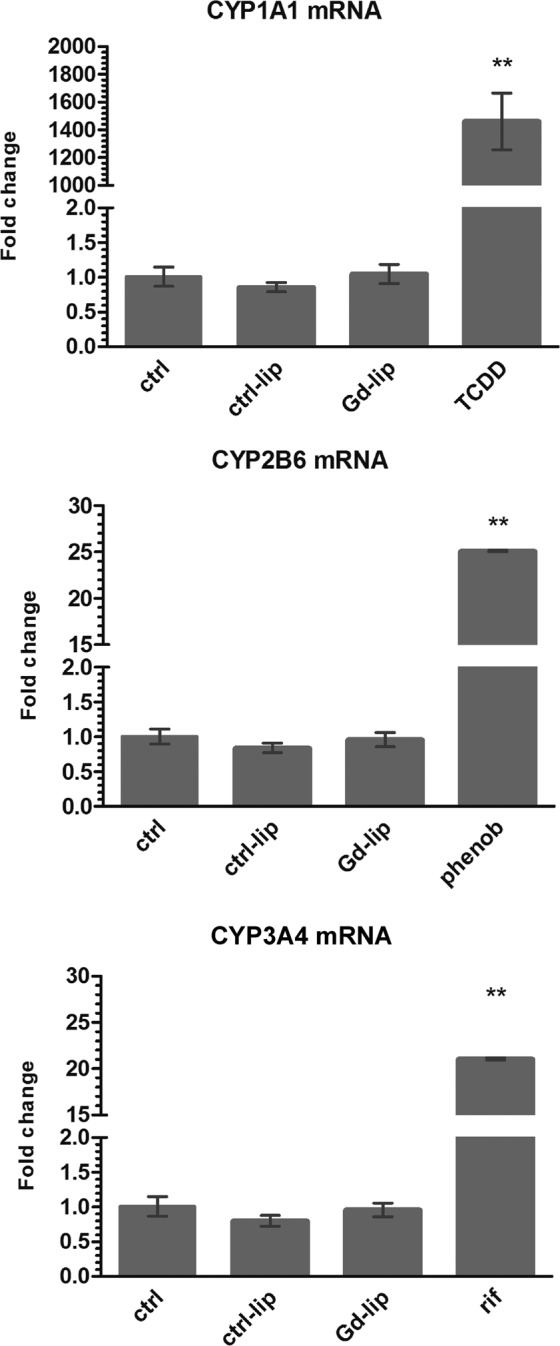
Induction of xenobiotic metabolizing enzyme genes CYP1A1, CYP2B6, and CYP3A4. Differentiated HepaRG cells were exposed to ctrl–lip or Gd–lip for 24 h and mRNA levels were determined by RT–PCR. 24h exposure to 1 nM 2,3,7,8-tetrachlorodibenzo-*p*-dioxin (TCDD), 1 mM phenobarbital (phenob) or 100 μM rifampicin (rif) were used as positive controls. The results are expressed as means ± s.d. of four independent experiments. The means are significantly different from negative control at **p < 0.01.

In liver and other organs, adaptive and toxic responses to xenobiotics are coordinated by xenobiotic receptors (XRs)^20^. Activation of XRs leads to increased transcription of xenobiotic metabolizing enzymes, such as cytochromes P450 (CYP). In order to determine if Gd–lip can affect XRs, we measured expression of specific genes CYP1A1, CYP2B6 and CYP3A4, which are under the transcription control of major XRs in differentiated HepaRG cells. Neither Gd–lip nor ctrl–lip increased mRNA levels of CYP1A1, CYP2B6 and CYP3A4 (Fig. 7), suggesting no XRs activation.

### Gd–liposomes do not induce activation of inflammasome

**Figure 8:**
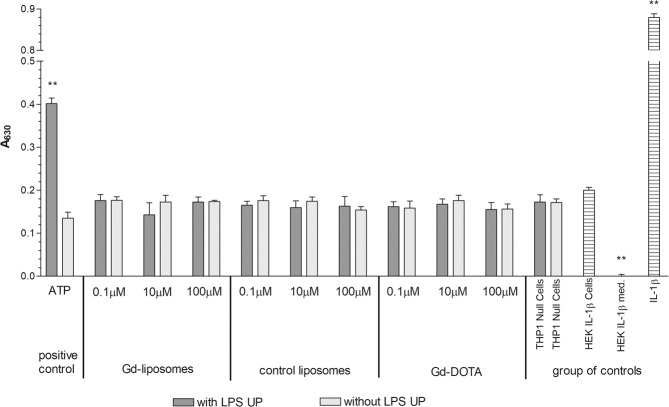
Stimulation of NLRP3 receptor by Gd–lip, ctrl–lip and Gd–DOTA. Testing system based on THP1–Null Cells and HEK–Blue™ IL–1β Cells (InvivoGen) was used for determination of NLRP3 receptor activation. Adenosine triphosphate (ATP) and recombinant interleukin 1β (IL–1β) were used as positive controls. The results are expressed as means ± s.d. of three independent experiments. The means are significantly different from negative control (control untreated HEK–Blue™ IL–1β Cells and THP1–Null cells) at **p < 0.01. Abbreviations: LPS UP: lipopolysaccharide ultrapure.

Finally, pro–inflammatory effects of Gd–lip were studied. Activation of inflammasome NLRP3 was measured by detecting the production of IL–1β from activated macrophage–derived THP1–Null cells, exposed to ctrl–lip or Gd–lip (Fig. 8). The activation of IL–1 receptor was detected in HEK–Blue™ cells expressing a secreted alkaline phosphatase (SEAP) reporter gene, which is induced by NF–κB/AP–1 upon activation of the cytokine signalling cascade. Levels of SEAP in the supernatant were monitored using QUANTI–Blue™ (InvivoGen, France).

Additionally, the concentration of both pro–inflammatory and anti–inflammatory eicosanoids, released into the cultivation medium from HepaRG exposed to ctrl–lip and Gd–lip, were determined by LC/MS–MS (Supplementary Table S2). We found no changes in any tested parameters, which indicates that neither ctrl–lip nor Gd–lip induced activation of inflammasome in THP1 cells and production of pro–inflammatory cytokines in differentiated HepaRG.

## Discussion

Complex physical–chemical characterization of tested preparation is the main prerequisite for toxicological studies. Gd–lip were characterized by monomodal size distribution with low polydispersity. Association of Gd with liposomes and localization in the lipid bilayer were proven by cryo–TEM and EDX analysis. This finding is important because the interaction of Gd^+3^ with molecules of water is a prerequisite for reaching sufficient values of T1 relaxivity and therefore good MRI contrast^21,22^. This liposomal preparation exerted the value of T1 relaxivity above 6 mmol^−1^s^−1^ at 9.8 T (*unpublished results*). This value is favourable for possible future application in MRI imaging of thrombi. Therefore, this *in vitro* toxicological study was performed with a thoroughly characterized liposomal preparation to get consistent results.

Gd^+3^–containing liposomes have been studied as nanocarriers of MRI for non–invasive imaging and they exhibit a favourable platform for a new generation of targeted diagnostic and theranostic systems. Biocompatibility of liposomes together with their ability of active or passive targeting is the main advantage of the nanocarriers^5^. In spite of the fact that liposomes are well tolerated nanoparticles approved by the Food and Drug Administration (FDA) and European Medicines Agency (EMA) for certain application in humans, modification of liposomes by Gd^+3^ complexes introduces a potential risk because of the known toxicity of free Gd^+3^ cations. Binding of Gd^+3^ in chelating complexes with a high stability constant effectively suppresses toxicity of Gd^+3^. Contrast agents like Gadovist® (Gadobutrol based preparation) are used in preclinical and clinical MRI imaging. Some rare side effects are documented especially in patients tending to allergic reactions^23^. Gadobutrol is well tolerated and has a favourable safety profile for patients of all age groups. The most frequent side effects were found as anaphylactoid/hypersensitivity reactions, headache, nausea, dizziness, injection site reactions, feeling hot, vomiting and dysgeusia^24^. In an animal model, Gd–based contrast agents were shown to induce necrosis and apoptosis in liver^25^.

Nevertheless, entrapment of Gd^+3^ in nanoliposomes, as well as in other nanocarriers, can affect biodistribution and accumulation of Gd^+3^ in organs and change excretion of Gd^+3^ from the organisms. Therefore, a more detailed study of potential toxicological effects of nanoparticle–based Gd^+3^ formulations on different organs and cell types is of importance. In the case of Gd–lip, hepatocytes and immune cells represent the most important first line off–target cells affected by Gd^+3^ which can induce events leading to pathological consequences manifesting in acute or chronic toxicity symptoms.

A number of studies have explored different features of liposomes containing PE-DTPA (Gd) chelator lipid as MRI contrast agent to date^7,13,26,27^. In clinical practice, liposomal drugs and contrast agents are administered by *i*.*v*. infusion and nanoliposomes can escape from circulation through fenestration in the blood vessel endothelium. In the liver, liposomes can penetrate through fenestrations, openings in sinusoidal endothelial cells^28^. Therefore, hepatocytes, Kupffer cells and liver sinusoidal endothelial cells can be exposed to nanocarriers.

HepaRG cells differentiate into hepatocyte–like colonies surrounded by biliary epithelial–like cells^19^ and represent a liver model closest to primary hepatocytes. Compared to commonly used hepatic cell lines, HepaRG have better liver–specific functionality, such as CYP and nuclear receptor expressions^29^, and were shown to be a useful model for drug–induced human hepatotoxicity studies^30–32^. In this study, the HepaRG cells were used to screen possible *in vitro* toxicity of Gd–lip. Based on data mining approach, Zhang *et al*.^14^ suggested deregulation of four genes (EGR1, ATF3, GDF15 and FGF21) as a suitable set of markers of early toxicity. These genes were upregulated far before the toxicity was visible at the molecular or phenotypic level. The genes were identified in primary human and rat hepatocytes and can predict liver and kidney pathology *in vivo*^14^. EGR1 and ATF3 are multifunctional stress–induced transcription factors that can be induced by e.g. hypoxia, oxidative stress, ER stress, genotoxic stress and inflammation. The early response genes regulate many versatile cellular responses, such as cell cycle, metabolism, cell adhesion and inflammatory response^33–36^. GDF15 and FGF21 are endocrine factors associated with liver and metabolic diseases. Expression of GDF15 and FGF21 can be induced by multiple intracellular stress signals, e.g. ER stress, amino acid deficiency and oxidative stress^37–39^. In our study, no cytotoxicity of Gd–lip was observed up to 72 h and the four early toxicity markers were not activated. Therefore, the data are in a good accordance and confirmed the stability of Gd–complex incorporated in liposomes.

Gd–lip were shown to be internalized and accumulated in lysosomes. Various intracellular systems could be affected and potentially disturbed by applied nanoparticles, resulting in induction of cellular stress, e.g. in case of lysosomal malfunction. Cells undergoing stress activate protective mechanisms that help them to maintain homeostasis and therefore to survive. Heat shock and DNA damage response, UPR, response to oxidative stress and activation of xenobiotic metabolizing enzymes belong among the stress–induced cellular responses. However, if the stress is too strong, signalling cascades leading to cell death are activated^40^. The heat shock response is characterized by induction of HSPs by heat shock factor 1. HSPs help the refolding of proteins damaged by e.g. heat, oxidative stress and heavy metals. Stress–inducible HSPA1B covers the extra demand of chaperoning activity in the cytoplasm (for detailed review see^15,41,42^). DNA damage response mechanism is activated to maintain genome stability and include increased stability and transcription activity of p53 (for review see^18^). CDKN1A, the p53 target gene, has multiple roles in DNA damage response and may serve as one of biomarkers of chemical (and nanoparticle) toxicity^43^. UPR is a compensatory mechanism of ER stress that is induced by accumulation of misfolded and unfolded proteins in ER. Three branches of UPR may be activated after dissociation of binding immunoglobulin protein (BiP) from three distinct ER stress sensors, ER transmembrane proteins ATF6, PERK and IRE1, resulting in the increased transcription of genes involved in protein folding, unfolded protein degradation and cell fate regulation (for review see^16,44^). In our study, DDIT3, XBP1s and HSPA5 genes were selected as representative biomarkers of all three UPR branches. None of the tested stress–induced genes was activated by Gd–lip, which implies indifferentness and tolerability of these structures by the cells.

Oxidative stress is a frequent response of cells to foreign structures like nanoparticles. In cells, oxidative stress causes the disturbance in the balance between reactive oxygen species (ROS) production and antioxidant defence mechanisms. The excess of ROS damages nucleic acids, proteins, carbohydrates, and lipids, which may result in cell death^40^. Nuclear factor erythroid 2–related factor (Nrf2), belonging among oxidative stress activated factors, regulates the expression of protective antioxidant genes, such as HMOX–1^17^. Lipid peroxidation products, 8-iso-PGF_2α_, the arachidonic acid oxidation product, and HODEs, arisen from linoleic acid oxidation, may serve as biomarkers of oxidative stress both *in vivo* and *in vitro*^45^. Eicosanoids, including prostaglandins, thromboxanes, and hydroxyeicosatetraenoic acids, are enzymatic oxidation products of arachidonic acid. Eicosanoids are biologically active lipid mediators with implication for inflammation and carcinogenesis^46,47^. Our data demonstrated that genes related to oxidative stress were not induced and no changes were detected in concentration levels of proinflammatory eicosanoids. These data are in a good agreement with the results concerning the induction of inflammasome NLRP3 in THP1 cell line. Tissue macrophages in lungs, spleen and liver are the off–target cells because of scavenging liposomes from circulation. Bioresistant nanoparticles (e.g. inorganic or carbon–based nanoparticles) are internalized by macrophages and can activate inflammasomes triggering production of proinflammatory cytokines (IL–1, IL–18)^48–50^. The induction of local acute or chronic inflammation affects tissue and organ function^51^.

In the liver and intestine, toxic and protective responses after environmental chemical exposure are coordinated by XRs, among others cytoplasmic aryl hydrocarbon (AhR), nuclear constitutive androstane (CAR), and pregnane X receptor (PXR). Activation of XRs leads to increased transcription of xenobiotic metabolizing enzymes, e.g. cytochromes CYP1A1, CYP2B6, and CYP3A4^52^. Both induction and suppression of detoxifying cytochrome enzymes are markers of stress and *in vivo* are related to inflammation^53^. Gd–lip were not found to induce expression of CYP enzymes, which means that potential application *in vivo* will not change the activity of these enzymes in liver and will possibly not affect metabolic and detoxifying processes depending on cytochromes P450.

We set up a complex *in vitro* system for preclinical evaluation of possible toxic effects of drugs and nanoparticle based drug carriers. This system comprises the main regulatory and acting pathways responding to harmful cellular stress. Application of this system did not reveal any adverse effects of the tested nanoliposomal Gd^+3^ preparation on cells related to human hepatocytes and macrophages.

This study was based on complex *in vitro* tests sensitive for revealing stimulation of stress–induced pathways. Of course, there is also some limitation related generally to lower complexity of *in vitro* systems in comparison to *in vivo* ones represented by whole organ or entire body. But the findings related to stimulation of the main proinflammatory pathways imply that Gd^+3^ nanoliposomes will leave immune system silent also at *in vivo* conditions.

In conclusion, in our study, we set up a complex *in vitro* system for preclinical evaluation of possible toxic effects of drugs and nanoparticle based drug carriers. We showed that nanoliposomal Gd^+3^ had no adverse impact on human derived hepatocyte-like HepaRG cells and macrophages. This finding is important because it provides reassurance that further preclinical testing on animal model is justified.

## Methods

### Chemicals

18:0 PE-DTPA, 18:0 PE-DTPA (Gd), DSPC, and 18:1 Liss Rhod PE were purchased from Avanti Polar Lipids (Alabama, USA), Gd–DOTA from Macrocyclics (Texas, USA); BaP from AccuStandard (Connecticut, USA) and TCDD from Cambridge Isotope Laboratories (Massachusetts, USA). All other reagents were purchased from Sigma–Aldrich, if not indicated otherwise.

### Liposome preparation

Liposomes were prepared by hydration of lipid film method followed by extrusion through polycarbonate filters (pore size 100 nm). The 18:0 PE-DTPA was used as a Gd–chelating lipid. The composition of Gd–lip was DSPC/Cholesterol/PE-DTPA (Gd), 65/30/5% (molar % of lipid). Liposomes containing DSPC/Cholesterol/PE-DTPA (ammonium salt), 65/30/5% (molar % of lipid) were used as a negative control.

### Liposome characterization

Physical-chemical characterization was performed by dynamic light scattering and cryo-TEM methods. Concentration of phospholipids was measured by Stewart´s method. Detailed description of the methods can be found as Supplement S3.

### Identification of Gd in liposomes by TEM–EDX

Samples of Gd–lip were suspended within a drop of phosphate buffered saline (PBS). The resulting suspension was covered with a Cu grid (300 Old Mesh, Agar Scientific, Austria), coated with Formvar film and carbon. The grid was removed from the suspension after 1 min and the residual liquid was dried with a strip of filtration paper. No negative staining was used. Transmission electron microscope Jeol 2100 coupled with EDX (Silicon Lithium Detector; Oxford x-MAX 80T, UK) at accelerating voltage 160 and 200 kV was used for chemical analysis and detection of Gd in liposomes.

### Determination of Gd content using inductively coupled plasma optical emission spectrometer (ICP–OES)

Concentration of Gd was measured by ICP–OES Ultima 2 (Horiba Jobin Yvon, France) in the sample of liposomes at the concentration of 1 mg/ml. The Gd standard for ICP (1 g/l) was used as the calibration solution diluted from 0.1 to 50 mg/l. Measurement was carried out in a cyclone spray chamber with argon flow rates 13.96 and 0.78 l/min for plasma and auxiliary gas, respectively. Peristaltic pump speed rate was 15 rpm and generator output was 1,400 W. Emission characteristics were measured at wavelength of 342 and 246 nm.

### Cell culture

The cells were cultivated in plastic tissue culture plates or dishes (TPP, Switzerland) at 37°C in atmosphere of 5% CO_2_. The HepG2 cells (ATCC, VA, USA) were grown in Dulbecco’s modified Eagle (DMEM) medium (Gibco, Life Technologies Corporation, UK) supplemented with 10% fetal bovine serum (FBS), 24 mM NaHCO_3_, 10 mM HEPES and antibiotics. The human hepatic progenitor HepaRG^®^ cells (Biopredic, France) were cultivated in William’s E Media with GlutaMAX™ Supplement (Gibco, Life Technologies Limited, UK) enriched with 10% FBS, 5 µg/ml insulin, 50 µM hydrocortisone, and antibiotics. The differentiation was performed according to the manufacturer’s protocol. Briefly, HepaRG were seeded at the density of 20.000 cells/cm^2^ and grown for 14 days. Then, the cells started to differentiate in the presence of 2% DMSO. Both growth and differentiation medium were renewed every 2 to 3 days. After additional 14 days, two cell types, hepatocytes and primitive biliary epithelial cells, were clearly distinguishable^54^. At that time, the differentiation medium was exchanged for cultivation medium and after another 3 days the cells were exposed to fresh medium with Gd–lip or ctrl–lip for 24 h. THP1–Null Cells (derived from THP1 human monocytic cells; InvivoGen, France) were grown in suspension in RPMI 1640 medium supplemented with 10% heat inactivated FBS, 2 mM L-glutamine, and antibiotics.

### Cytotoxicity assay

Cytotoxicity was tested using NR uptake assay in liver cancer HepG2, progenitor HepaRG and differentiated HepaRG cells. The confluent cells, grown in 96 well tissue culture plates, were exposed to Gd–lip, ctrl–lip or low molecular weight complex Gd–DOTA for 24 h – 72 h. Subsequently, the exposition medium was removed and NR dissolved in medium (40 μg/ml) was added for 3 h. The cells were then fixed in 0.5% formaldehyde with CaCl_2_ for 15 min, washed twice in PBS and lysed by 1% acetic acid in 50% methanol. The absorbance was measured using multiplate reader Synergy II (BioTec, USA). The final concentration of Gd ranged between 1 μM – 100 μM. The concentration of ctrl–lip was calculated to have the same lipid concentration as Gd–lip.

### Confocal laser scanning microscopy

Confocal fluorescence was applied to study the liposomes localization in the differentiated HepaRG cells. The cells were seeded in glass bottom µ–Slide (Ibidi GmbH, Germany), differentiated and exposed to Gd–lip fluorescently labelled with 18:1 Liss Rhod PE for 4 h or 24 h. The final total lipid concentration was 25 μg/ml, which corresponded to 180 nM Gd. 1 h before life–cell imaging, the cells were incubated with 100 nM LysoTracker® Green DND-26 (Molecular Probes, OR, USA) and 2 ug/ml Hoechst 33342 (Enzo Life Sciences, NY, USA) to visualize lysosomes and nuclei, respectively. Life–cell imaging (37°C, 5% CO_2_) was performed with the TCS SP8 confocal microscope (Leica Microsystems, Germany).

### Sphingolipid analysis

Differentiated HepaRG cells, exposed to Gd–lip or ctrl–lip (total lipid concentration 25 μg/ml) for 24 h, were washed twice with PBS and harvested in ice–cold methanol (1.5 ml). Subsequently, chloroform (0.75 ml) was added to the cell suspension. After sonication, extraction process continued at laboratory temperature overnight. Solvents were then removed by nitrogen drying and the residue was reconstituted in methanol/chloroform mixture (3:1 ratio). Sphingolipid species were separated by reverse high–performance liquid chromatography (HPLC; Dionex Ultimate 3000; Thermo Scientific) and detected by MS–MS, using multiple reaction monitoring (MRM) scan mode (QTRAP 4500 with ESI; AB Sciex, Canada). HPLC conditions were as follows: GEMINI column C18 250x4.6 mm (Phenomenex, USA), flow rate 0.7 ml/min., gradient mobile phases RA/RB (RA = methanol/water 60/40, RB=methanol; starting ratio RA/RB 60/40, total time 69 min). MS–MS conditions were as follows: ESI in positive mode, drying air, fragmentor voltage and collision energy voltage optimized for each sphingolipid species.

### Real–time quantitative RT–PCR

The differentiated HepaRG cells grown in 24 well plates were exposed to Gd–lip or ctrl–lip (total lipid concentration 25 μg/ml) for 24 h. Thapsigargin, tunicamycin, BaP, TCDD, phenobarbital, rifampicin and H_2_O_2_ were used as positive controls. The cells were washed twice with PBS and harvested into the cell lysis buffer provided in the NucleoSpin RNA II Purification Kit (Macherey Nagel, Germany). Total RNA was isolated according to the manufacturer’s instructions. The levels of ATF3, CDKN1A, CYP1A1, CYP2B6, CYP3A4, DDIT3, EGR1, FGF21, GDF15, HMOX1, HSPA1B, HSPA5, and XBP1s mRNAs were determined by real–time RT–PCR. The sequences of primers and probes used for detection of GDF15, CYP1A1, CYP2B6, CYP3A4 and XBP1s mRNA were described previously^55–57^. The sequences of the other primers, TaqMan probes (all designed and synthesized by Generi Biotech, Czech Republic), and numbers of employed Universal Probe Library probes (UPL; Roche Life Sciences, Germany) are listed in Supplementary Table S4. Hydroxymethylbilane synthase (HMBS; NM_000190) was used as the reference gene (predesigned qPCR assay, 3032-F, Generi–Biotech). The amplifications were carried out in 10 µl reaction mixture containing: 5 µl of QuantiTect Probe RT–PCR Master Mix, 0.1 µl QuantiTect RT mix (Qiagen GmbH, Germany), 1.1 µl solution of primers and probe, 1.8 µl water and 2 µl total RNA sample and were run on the LightCycler® 480 System (Roche Diagnostics, Czech Republic) using the program published previously^58^. Changes in gene expression were calculated using the comparative threshold cycle method^59^.

### Activation of NLRP3 (Inflammasome)

Production of IL–1β was detected with HEK–Blue™ IL–1β Cells (InvivoGen, France) according to the manufacturer’s instructions. Briefly, Gd–lip, ctrl–lip or Gd–DOTA were added to the suspension of THP1–Null Cells activated by lipopolysaccharide (LPS). After 24 h, THP1 supernatant was added to the suspension of HEK–Blue cells and incubated overnight. Activation of IL–1 receptor was detected by monitoring of the activation of the SAEP reporter gene using QUANTI–Blue™ and quantified by Synergy II spectrophotometer. Recombinant IL–1β (InvivoGen) and ATP were used as positive controls. For more detailed description of the method see^60^.

### Arachidonic acid metabolites

The amount of arachidonic acid metabolites, eicosanoids, released into the cultivation medium was measured as described previously^61^. Briefly, after the 24h exposure of differentiated HepaRG cells to ctrl–lip or Gd–lip, the medium was harvested, extracted using solid–phase–extraction and the concentration of eicosanoids was measured by LC/MS–MS. All eicosanoid standards were purchased from Cayman Chemical Company (USA).

### Statistical analysis

The results were expressed as means ± s.d. The data were analyzed by Shapiro–Wilk normality test, one-way ANOVA and Dunnett’s multiple comparison test or unpaired Student’s t-test using GraphPad Prism 7.04.

## Supplementary information

Supplementary information.

## Data Availability

The datasets used and/or analysed during the current study are available from the corresponding authors on reasonable request.
