# Supplementary material for: Gadolinium labelled nanoliposomes as the platform for MRI theranostics: in vitro safety study in liver cells and macrophages
Source: Sci Rep. 2020 Mar 16;10:4780. doi: 10.1038/s41598-020-60284-z (PMC7075985; doi:10.1038/s41598-020-60284-z)
Supplement: Supplementary file 1 — Supplementary information. [file 41598_2020_60284_MOESM1_ESM.pdf]

Supplementary information

Gadolinium labelled nanoliposomes as the platform for MRI theranostics: *in vitro* safety study in liver cells and macrophages

Pavlína Šimečková[1], František Hubatka[1], Jan Kotouček[1], Pavlína Turánek Knötigová[1], Josef Mašek[1], Josef Slavík[1], Ondrej Kováč[1], Jiří Neča[1], Pavel Kulich[1], Dominik Hrebík[2], Jana Stráská[3], Kateřina Pěnčíková[1], Jiřina Procházková[1], Pavel Diviš[4], Stuart Macaulay[5], Robert Mikulík[6,7], Milan Raška[1,8], Miroslav Machala*[1], Jaroslav Turánek*[1].

1. *Veterinary Research Institute, Brno, Czech Republic*
2. *Central European Institute of Technology CEITEC, Structural Virology, Masaryk University, Brno, Czech Republic*
3. *Regional Centre of Advanced Technologies and Materials, Palacký University, Olomouc, Czech Republic*
4. *Faculty of Chemistry, Technical University, Brno, Czech Republic*
5. *Malvern Instruments, Great Malvern, UK*
6. *International Clinical Research Centre, St. Anne's University Hospital Brno, Czech Republic*
7. *Neurology Department, St. Anne's University Hospital and Masaryk University, Brno, Czech Republic*
8. *Department of Immunology, Faculty of Medicine and Dentistry, Palacký University, Olomouc, Czech Republic*

*** Corresponding authors:**

Res. et Ass. Prof. RNDr. Jaroslav Turánek, Res. Prof.

Department of Pharmacology and Immunotherapy

Veterinary Research Institute, Hudcova 70, 621 00 Brno, Czech Republic

E–mail: turanek@vri.cz, Phone: +420 533 331 311

RNDr. Miroslav Machala, CSc.

Department of Chemistry and Toxicology

Veterinary Research Institute, Hudcova 70, 621 00 Brno, Czech Republic

E–mail: machala@vri.cz, Phone: +420 533 331 801

Supplementary figure S1: Neutral red cytotoxicity assay.

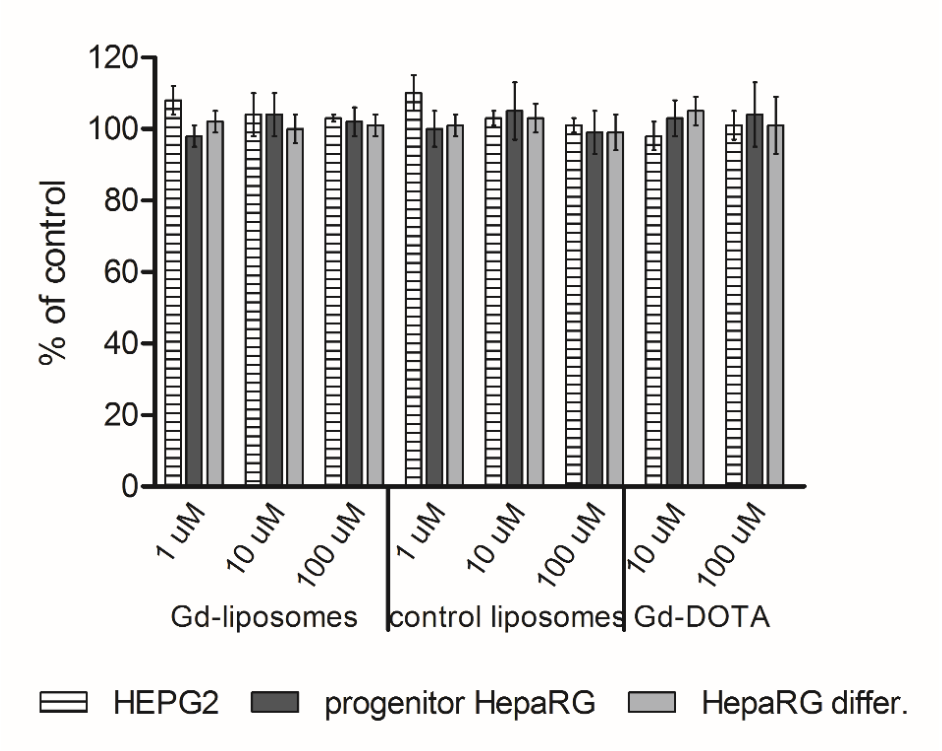

HepG2, HepaRG undifferentiated (progenitor) and differentiated (differ.) HepaRG were exposed to Gd–liposomes, control liposomes or Gd–DOTA for 72 h. The concentration of Gd was 1 μM, 10 μM and 100 μM. Total lipid concentrations of Gd-liposomes and control liposomes were of the same values. Values are presented as means ± s.d. of three independent experiments. No statistically significant changes compared to control cells were detected.

Supplementary table S2: Changes of eicosanoid concentrations in cell culture medium after exposure of differentiated HepaRG cells to ctrl–lip or Gd–lip for 24 h, measured by LC/MS-MS.

a

	6-keto-PGF1α	TxB2	PGF2α	PGE2	PGD2	15-keto-PGE2	PGF2β
control	1±0.3	1±0.04	1±0.1	1±0.2	1±0.2	1±0.1	1±0.2
ctrl-lip	0.9±0.7	1.0±0.1	0.7±0.2	0.8±0.1	0.8±0.1	1.0±0.4	1.1±0.5
Gd-lip	0.9±0.5	0.9±0.05	0.9±0.1	0.9±0.2	1.1±0.3	0.9±0.5	0.9±0.2

b

	LXA4	13,14-DH-15-keto-PGE2	13,14-DH-15-keto-PGD2	PGA2	PGJ2
control	1±0.1	1±0.1	1±0.4	1±0.3	1±0.4
ctrl-lip	0.8±0.3	1.0±0.1	0.7±0.2	0.9±0.3	1.0±0.4
Gd-lip	0.8±0.2	0.9±0.2	0.7±0.4	0.8±0.1	0.9±0.3

c

	20-HETE	15-HETE	11-HETE	8-HETE	12-HETE	9-HETE	5-HETE	AA
control	1±0.1	1±0.1	1±0.1	1±0.08	1±0.2	0.9±0.4	1±0.2	1±0.04
ctrl-lip	1.0±0.4	0.7±0.2	0.8±0.1	0.8±0.2	0.8±0.1	0.8±0.1	1.1±0.4	0.9±0.06
Gd-lip	0.8±0.3	0.9±0.2	1.1±0.2	0.7±0.2	0.9±0.1	1.3±0.9	1.1±0.4	0.9±0.09

Multiples of control concentration (values in medium of untreated cells) of prostaglandins (PG), thromboxane (TX) and leukotrienes (LX) (Table **a, b**), HETEs and arachidonic acid (AA; Table **c**) are expressed as mean ± s.d. of four independent experiments. No statistically significant changes compared to control cells were found.

Supplement S3: Liposome characterization

Measurement of the size of Gd–liposomes and calculation of particle concentration

The size distribution, Z–average diameter and polydispersity index (PDI) were obtained from the autocorrelation function using a Zetasizer Ultra (Malvern Panalytical, UK) equipped with 633 nm HeNe laser and operating at detection angle of 173° and at controlled temperature of 25°C. The ζ–potential was measured in 10 mM Tris, pH 7.4. Number of particles was calculated using MADLS (multiangle dynamic light scattering) mode and software ZS Explorer version 1.2.0.91. For calculation of the number of particles, samples of liposome suspension (1 mg of total lipid/ml) were diluted 1:10. The diluted suspension was placed in a disposable, low volume cuvette with path length 10 mm (Malvern Panalytical, UK).

Analysis of phospholipids by Stewart´s method

The final concentration of phospholipids in the liposomal samples and in the lipid extracts was determined for each preparation according to Stewart method based on quantification of the complex formed by phospholipid with ammonium ferrothiocyanate in the organic solution.

Cryo–TEM sample preparation and micrograph acquisition

Prior to sample vitrification, quantifoil grids (R2/1, mesh 300 grid) were cleaned in plasma cleaner (Quorum Technologies) by exposure to plasma for 15 s. The samples for cryo–TEM analyses were prepared by applying liposome suspension (3.8 μL, 1 mg/ml) onto a grid, blotted, and vitrified by rapid plunging into liquid ethane using a Vitrobot Mark IV (FEI) which was operated at 20°C, 100% humidity, blot time 2 s, blot force -2. The grids were then stored in liquid nitrogen. Micrographs were acquired using a Tecnai F20 (FEI) microscope operated at 200 kV, equipped with cryo–holder (Gatan, Inc.) and Eagle CCD camera (FEI). The microscope was aligned to achieve parallel electron beam and dose was calibrated to ~22 e^-/ ($Å^2$ s). Nominal defocus was set to ~3.5 μm.

Supplementary table S4: List of sequences of primers and UPL probe numbers used in RT-PCR.

Gene symbol / RefSeq code		Sequences
ATF3	NM_001206486.2<br>NM_001206488.2<br>NM_001040619.2<br>NM_001030287.3<br>NM_001674.3<br>NM_001206484.2	F: 5'-TTTGCCATCCAGAACAAGC-3'<br>R: 5'-CATCTTCTTCAGGGGCTACCT-3'<br>P: #53
CDKN1A	NM_000389.4	F: 5'-CCGAAGTCAGTTCCTTGTGG-3'<br>R: 5'-CATGGGTTCTGACGGACAT-3'<br>P: #82
DDIT3	NM_001195056.1<br>NM_001195054.1<br>NM_001195053.1<br>NM_001195055.1<br>NM_004083.5<br>NM_001195057.1	F: 5'-AAGGCACTGAGCGTATCATGT-3'<br>R: 5'-TGAAGATACACTTCCTTCTTGAACA-3'<br>P: #21
EGR1	NM_001964.2	F: 5'-AGCCCTACGAGCACCTGAC-3'<br>R: 5'-GGTTTGGCTGGGGTAACTG-3'<br>P: #22
FGF21	NM_019113.3	F: 5'-ATGGGGCCCTGTATGGAT -3'<br>R: 5'-AAACATTGTATCCGTCCTCAAGA -3'<br>P: #24
HMOX1	NM_002133.2	F: 5'-AGACTGCGTTCCTGCTCAAC -3'<br>R: 5'-GGCTCTGGTCCTTGGTGTC -3'<br>P: #17
HSPA1B	NM_005346.4	F: 5'-CTCCGACCTGTTCCGAAG -3'<br>R: 5'-GAATCTGGGCCTTGTCCA-3'<br>P: #1
HSPA5	NM_005347.4	F: 5'-AGCCTGGCGACAAGAGTG-3'<br>R: 5'-TCCTTGGGCAGTATTGGATT-3'<br>P: #39
